# Supplementary material for: Hybrid Models and Biological Model Reduction with PyDSTool
Source: PLoS Comput Biol. 2012 Aug 9;8(8):e1002628. doi: 10.1371/journal.pcbi.1002628 (PMC3415397; doi:10.1371/journal.pcbi.1002628)
Supplement: Text S4 — Complete source code for the PyDSTool package (version 0.88.120504). Includes API documentation and help files linking to web pages. This file is identical to the current public release on Sourceforge.net. (ZIP) [file pcbi.1002628.s004.zip › PyDSTool/html/PyDSTool.Toolbox.optimizers.line_search.wolfe_powell_rule-pysrc.html]

xml version="1.0" encoding="ascii"?


PyDSTool.Toolbox.optimizers.line\_search.wolfe\_powell\_rule


| Home | Trees | Indices | Help | PyDSTool |
| --- | --- | --- | --- | --- |

Package PyDSTool ::
Package Toolbox ::
Package optimizers ::
Package line\_search ::
Module wolfe\_powell\_rule

[hide private]

[frames] | no frames]

# Source Code for Module PyDSTool.Toolbox.optimizers.line\_search.wolfe\_powell\_rule

```
 1   
 2  # Matthieu Brucher 
 3  # Last Change : 2007-08-22 14:05 
 4   
 5  import numpy 
 6   


7 -class WolfePowellRule(object):


8    """ 
 9    The standard Wolfe-Powell rule for a inexact line search 
10    """ 


11 -  def __init__(self, alpha = 1., rho = 0.1, sigma = 0.4, alpha_min = 0., alpha_max = 1., alpha_limit = 0.1, **kwargs):


12      """ 
13      Initializes the search 
14      Parameters : 
15        - alpha is the first step size that will be tried (1.) 
16        - rho is the rhos acceptation factor (0.1) 
17        - sigma is the factor for the Wolfe-Powell rule (0.4) 
18        - alpha_min is the inf limit of the search interval (0.) 
19        - alpha_max is the max limit of the search interval (1.) 
20        - alpha_limit is a factor so that the estimated alpha is not near the limts of the tested bracket, leading to a divergence in the algorithm (alpha_limit = 0.1) 
21      Those parameters should be tweaked depending on the function to optimize 
22      """ 
23      self.alpha = alpha 
24      self.rho = rho 
25      self.sigma = sigma 
26      self.alpha_min = alpha_min 
27      self.alpha_max = alpha_max 
28      self.alpha_limit = alpha_limit

29   


30 -  def __call__(self, origin, function, state, **kwargs):


31      """ 
32      Tries to find an acceptable candidate 
33      """ 
34      direction = state['direction'] 
35      ak = self.alpha_min 
36      bk = self.alpha_max 
37      if 'initial_alpha_step' in state: 
38        alpha = state['initial_alpha_step'] 
39      else: 
40        alpha = self.alpha 
41      gradient = state['gradient'] 
42   
43      f1temp = function(origin) 
44      f1ptemp = numpy.dot(gradient, direction) 
45      while(True): 
46        #print ak, bk, alpha 
47        if numpy.isnan(alpha): 
48          state['alpha_step'] = 0 
49          return origin 
50   
51        ftemp = function(origin + alpha * direction) 
52        #First rule test 
53        if ftemp <= function(origin) + self.rho * alpha * numpy.dot(gradient, direction): 
54          fptemp = numpy.dot(function.gradient(origin + alpha * direction).T, direction) 
55          #Second rule, Wolfe-Powell, test 
56          if fptemp >= self.sigma * numpy.dot(gradient, direction): 
57            state['alpha_step'] = alpha 
58            return origin + alpha * direction 
59          else: 
60            alphap = alpha + (alpha - ak) * fptemp / (f1ptemp - fptemp) 
61            ak = alpha 
62            alpha = alphap 
63            f1temp = ftemp 
64            f1ptemp = fptemp 
65        else : 
66          bracketSize = abs(bk - ak) 
67          alphap = ak + (alpha - ak) / (2. * (1. + (f1temp - ftemp) / ((alpha - ak) * f1ptemp))) 
68          bk = alpha 
69          if abs(alphap - ak) < self.alpha_limit * bracketSize: 
70            alpha = ak + self.alpha_limit * bracketSize 
71          else: 
72            alpha = alphap

73
```

  


| Home | Trees | Indices | Help | PyDSTool |
| --- | --- | --- | --- | --- |

|  |  |
| --- | --- |
| Generated by Epydoc 3.0.1 on Fri May 4 15:24:18 2012 | http://epydoc.sourceforge.net |
